# ForametCeTera, a novel CT scan dataset to expedite classification research of (non-)foraminifera

**DOI:** 10.1038/s41597-024-03476-w

**Published:** 2024-06-17

**Authors:** Joost Luijmes, Tristan van Leeuwen, Willem Renema

**Affiliations:** 1 Naturalis Biodiversity Center, Marine Biodiversity Group, Darwinweg 2, 2333 CR Leiden, The Netherlands; 2 Centrum Wiskunde en Informatica, Computational Imaging Group, Science Park 123, 1098 XG Amsterdam, The Netherlands; 3 Delft University of Technology, Mekelweg 4, 2628 CD Delft, The Netherlands; 4 Utrecht University, Mathematical Institute, Budapestlaan 6, 3584 CD Utrecht, The Netherlands; 5 Institute for Biodiversity and Ecosystem Dynamics, University of Amsterdam, Science Park 904, 1098 XH Amsterdam, The Netherlands

**Keywords:** Marine biology, Climate-change ecology, Biodiversity, Microbial biooceanography, Bioinformatics

## Abstract

This paper introduces ForametCeTera, a pioneering dataset designed to address the challenges associated with automating the analysis of benthic foraminifera in sediment cores. Foraminifera are sensitive sentinels of environmental change and are a crucial component of carbonate-denominated ecosystems, such as coral reefs. Studying their prevalence and characteristics is imperative in understanding climate change. However, analysis of foraminifera contained in core samples currently requires washing, sieving and manual quantification. These methods are thus time-consuming and require trained experts. To overcome these limitations, we propose an alternative workflow utilizing 3D X-ray computational tomography (CT) for fully automated analysis, saving time and resources. Despite recent advancements in automation, a crucial lack of methods persists for segmenting and classifying individual foraminifera from 3D scans. In response, we present ForametCeTera, a diverse dataset featuring 436 3D CT scans of individual foraminifera and non-foraminiferan material following a high-throughput scanning workflow. ForametCeTera serves as a foundational resource for generating synthetic digital core samples, facilitating the development of segmentation and classification methods of entire core sample CT scans.

## Background & Summary

Benthic foraminifera are unicellular organisms characterised by a calcium carbonate shell. They are responsible for about 20% of global carbonate production^1^. In carbonate-dominated environments, such as coral reefs, foraminifera are important contributors to the production of sediment^2,3^. Combined effects of global climate change impair carbonate production in these environments^4–6^, which is critical for low-lying tropical islands to withstand sea-level rise^7,8^. Foraminifera are also sensitive sentinels of environmental change^9^. As such, they act as proxies to infer data on both long and short-temporal scales. For example, the species composition of an assemblage of foraminifera is related to environmental change^10,11^, thus analysing the gradient of such a composition by sampling over time^12^ or by observing a sediment core^13^, shows temporal trends in habitat quality. Monitoring foraminifera is thus imperative in improving our understanding of the response of these sensitive systems to climate change.

**Fig. 1:**
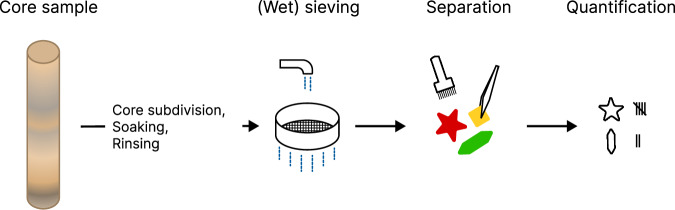
The conventional procedure of quantifying foraminifera. A core sample is subdivided, soaked in a (chemical) solution and rinsed, followed by (wet) sieving, separation, and classification and quantification of the species. The latter two steps are performed under a microscope.

Currently, collected sediment cores are washed and sieved to separate the foraminifera from the surrounding material. This is followed by manual partitioning, identification and quantification of the foraminifera (see Fig. 1). This is a time-consuming process that requires expert knowledge. As such, there has been active research in automating (parts of) this procedure. Such methods involve taking photographs of sieved and washed foraminifera and using machine learning to subsequently classify the imaged foraminifera. This is done by defining hand-crafted features^14^ or by learning the relevant features for classification based on the whole image of individual foraminifera^15–17^. Several efforts have incorporated 3D features by photographing specimens under different lighting conditions^14^, at different focal planes^18^ or both^17^. Despite these advances in automation, preparation of the sediment core sample (e.g. washing, sieving and sorting) remains a bottleneck. Moreover, foraminifera stuck in hardened consolidated sediment are difficult to obtain and classify in this manner^16,18^.

**Fig. 2:**
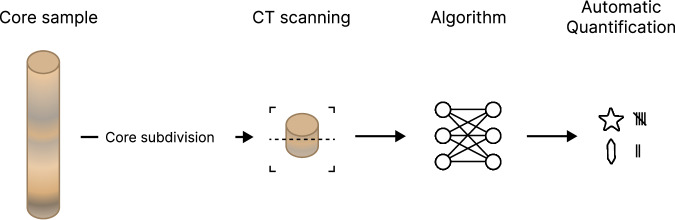
The proposed procedure of quantifying foraminifera. A core sample is subdivided and CT scanned. An algorithm then quantifies the 3D CT reconstruction.

We envision an alternative workflow, in which 3D X-ray computational tomography (CT) is used to further automate the procedure. While CT scanning has been widely employed to analyse *individual* foraminifera^19–25^, our workflow is based on scanning the whole core *before* washing and sieving. The resulting 3D image can then be processed digitally to separate the individual foraminifera contained within the core sample and classify them using advanced 2D and 3D machine learning techniques. A schematic depiction of the envisioned procedure is shown in Fig. 2.

The methods needed to segment and classify individual foraminifera from such 3D scans do not exist yet. To enable the development of such methods, high-quality labelled training data are needed. While there exist some datasets of CT scans of (individual) foraminifera species^19,20,26^, they are not suitable for developing and training methods for separating and classifying CT scans of whole core samples, as the data is lacking in magnitude, diversity and consistency. One recent, notable CT scan dataset^25^ improves in terms of (planktic) species diversity and dataset magnitude but still lacks in size for training machine learning algorithms. We aim to address these shortcomings with ForametCeTera; a dataset consisting of 436 3D CT scans of 288 individual benthic foraminifera and 148 bits of non-foraminiferan material. ForametCeTera’s data can be used as building blocks to generate synthetic digital core samples, which in turn can be used to develop methods for segmenting and classifying CT scans of core samples. We also demonstrate a high-throughput, specimen-agnostic scanning procedure suitable for the aforementioned task which can be used to rapidly build upon the dataset’s breadth and depth with additional microfossils. It is the first time that a dataset of foraminifera of this scope has been collected. We believe it will be helpful for method development and validation.

The remainder of the paper is organised as follows. A detailed description of how the dataset was acquired is given in the Methods section. This is followed by a detailed description of the dataset itself, how it was validated, and how it can be used.

## Methods

**Fig. 3:**
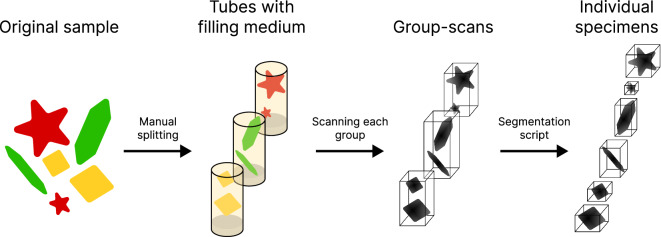
The specimen scanning procedure. From left to right, a sample of different specimens is split into groups by type and put in tubes with a filling medium, each tube is scanned following the same protocol, each group-scan is segmented producing density volumes of individual specimens.

To create the dataset, we carefully selected samples of individual foraminifera and bits of non-foraminiferan material. We will refer to either individual foraminifera or non-foraminiferan material as *specimens*. The specimens were sourced from core samples acquired by the Naturalis Biodiversity Center and split up into foraminifera species and a residual group of non-foraminiferan material. Each collection of specimens was put in a tube with a filling medium. Each tube was then CT scanned (creating a *group-scan*) with consistent scanning parameters deemed most suitable after performing trial scans of all specimens. A summary of this process is shown in Fig. 3. ForametCeTera contains 11 group-scans, that were segmented into a total of 436 individual specimens; 288 foraminifera and 148 bits of non-foraminiferan material. This process took place over a period of 2 months. The scope of the dataset encapsulates the raw CT projections of the group-scans, reconstructed 2D cross-sectional data of the group-scans, reconstructed 3D group-scans, segmented 3D specimens and all scripts that were used throughout this process.

### Sample selection

**Fig. 4:**
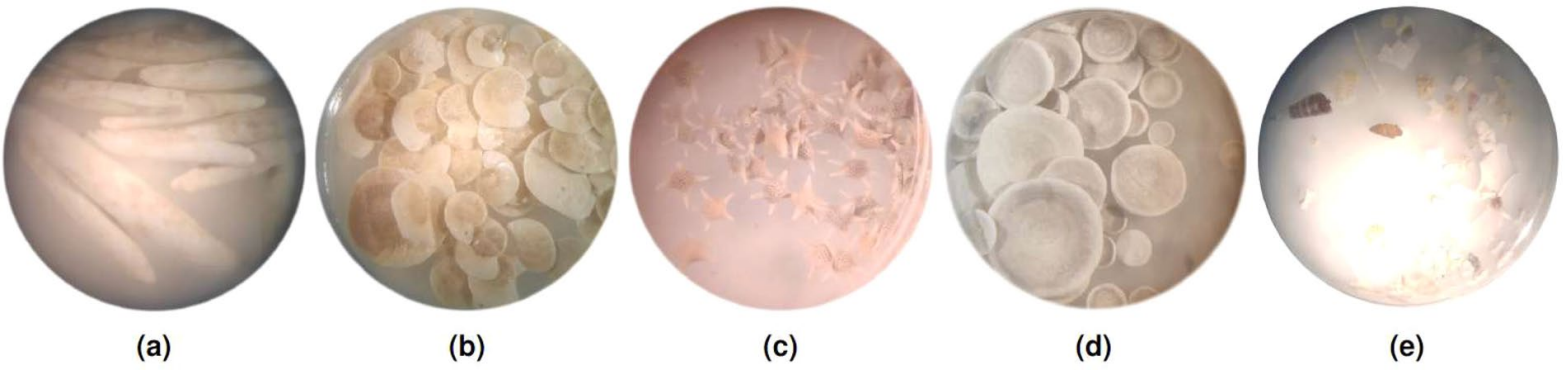
Microscope images of the selected samples. **(a) ***Alveolinella quoyi*. **(b) ***Operculina ammonoides*. **(c) ***Baculogypsinoides sphaerulata*. **(d) ***Marginopora santoensis*. **(e)** *γ*, an example of non-foraminiferan material.

The samples from which the specimens were retrieved, were collected in Makassar, Indonesia and Espiritu Santo, Vanuatu. A detailed overview of their metadata is shown in Table 1. These specimens have previously been used in academic and educational activities and have therefore been cleaned. However several specimens had clumps of sand sticking to them. Taking care to keep sandy samples separate, all specimens were split and grouped by their respective species and region. Non-foraminiferan material was separated too. The resulting collection of specimens is described in Table 2. Several microscope images of these specimens are shown in Fig. 4.

**Table 1:** Metadata of the samples from which the specimens were retrieved.

Index	Country	Area	Island	Sample	Latitude	Longitude	Depth	Date [y-m-d]
**A**	Indonesia	Makassar	Barang Caddi	UPG84zand2	5′04′40.8″S	119′19′00.8″E	25 m	2018-04-30
**B**	Indonesia	Makassar	Barang Caddi	UPG84zand2	5′04′40.8″S	119′19′00.8″E	25 m	2018-04-30
**C**	Vanuatu	Espiritu Santo	Aore	FR09-0,5	15′32′23.6″S	167′12′51.5″E	0.5 m	2016-10-15

The ‘Index’ column may be used to cross-reference the specimens’ origin between Table 2 and this Table.

**Table 2:** An overview of the scanned specimens.

Dataset ID	Metadata index	Species	No. specimens in group-scan	Size of group-scan .nrrd file [MB]	No. segmented specimens	Sum of specimens .nrrd files size [MB]
A1.2	A	*Alveolinella quoyi*	30	253	27	22
A2.2	A	*Alveolinella quoyi*	10	126	10	7.3
B1.3	A	*Operculina ammonoides*	50	211	48	5.4
B2.2	A	*Operculina ammonoides*	50	220	43	5.6
B3.2*	A	*Operculina ammonoides*	20	240	20	4.2
C1.2	C	*Marginopora santoensis*	50	328	47	19
D1.2	C	*Baculogypsinoides sphaerulata*	50	107	45	4.1
D2.2	C	*Baculogypsinoides sphaerulata*	50	93	48	3.6
*γ*1.2	C	Non-foraminiferan material	55	109	55	2.2
*θ*1.2	B	Non-foraminiferan material	50	179	50	11
*θ*2.2	B	Non-foraminiferan material	50	295	43	14

*Sandy sample.

The number of segmented specimens is generally lower than the initial number of specimens placed inside the tube due to instances where two touching specimens were erroneously segmented as one. Such instances were discarded. Details about specimen origin can be found using the ‘Metadata index’ column to index into Table 1.

### Sample preparation

In our testing, it was found that an adequate CT scan takes at least half an hour. It would be very time-consuming to perform such a scan for each individual specimen. As such, it was favoured to scan several specimens at once, creating the previously mentioned group-scans. This group-scan can then be segmented to obtain individual specimens. The successful application of segmentation algorithms requires the specimens to be adequately spaced. To achieve this, a filling medium was needed that fulfilled several requirements; 1) having minimal overlap in density to the specimens, 2) being relatively homogeneous and 3) given the value and utility of foraminifera, being separable from the specimens when dismantling the samples. The candidate filling media were sugar and coffee creamer as they both dissolve in water (requirement 3) and are readily available. After creating scans with both media, sugar was found to be both less homogeneous and have density overlap with the specimens. Subsequently, coffee creamer was the selected filling medium.

For each group-scan, the selected specimens and filling medium were put in a plastic tube (11.6 mm inner diameter, 50.7 mm inner height, 1.5 mm thick) with a screw cap. Specimens at the bottom of the tube are at risk of being poorly captured as they are near the thicker bottom of the tube and mounting equipment. As such, a 2 cm cylindrical piece of Styrofoam was first placed in the tube, raising the tube contents from the bottom. Subsequently, a teaspoon of filling medium was dispensed on a piece of paper with a crease through the middle. The specimen of choice was then disposed onto the filling medium and mixed with it. The mixed medium and specimen materials were poured into the tube following the aforementioned crease. Upon X-ray inspection, it may turn out that the specimens are poorly distributed throughout the filling medium. In such cases, simply shaking the tube can effectively redistribute the specimens throughout the medium.

### Scanner setup and parameters

To perform the CT scans, a Neoscan N80 FP micro-CT scanner was used, located at the Naturalis Biodiversity Center. The scanner contains a microfocus X-ray source (limited to 110 kV, 16 W), an active pixel CMOS flat-panel X-ray detector (7 Mp) and, in between these, a stage where the sample is mounted, capable of axial rotation and 3D translation^27^.

The CT scanner is operated using Neoscan’s accompanying software: Neoscan80, version 3.0.2. All scans were performed with a resolution of 15 *μ*m, using a 0.5 mm aluminium (Al) filter. Testing revealed that a different filter may enhance the image contrast of some specimens as, from certain angles, X-rays were too attenuated by the filter and the specimens. However, as one of the downstream aims of ForametCeTera is to generate synthetic samples, scanning with different parameters would introduce per-specimen biases. This particular filter turned out to be suitable for most specimens.

Prior to each scan, the CT scanner flat-field was automatically calibrated using the Neoscan80 software. For each scan, the object made a full 360° rotation with images captured at 0.2° increments resulting in 1801 projections, including a final overlapping projection. The exposure time was 94 ms and 4 averaging frames were used. The X-ray source was set to 67 kV and 200 *μ*A. Projections were captured at the highest possible resolution of 2400 × 2752 pixels. In case the region of interest exceeded the vertical field of view of the scanner, multiple scans were ‘stitched’ together using the oversize scanning feature of Neoscan80.

### Data acquisition

The projection data which resulted from the group-scans was reconstructed to create 2D cross-sectional images using the Neoscan80 software. Only intensity values between 0.09 and 0.9 were retained. These values are of a unitless quantity proportional to material density. The 2D cross sections were exported as a stack of lossless-compressed 8-bit .png files. These stacks were converted into lossless group-scan 3D data by means of a Python script (stack.py, see Code availability), producing .nrrd files, a file format for *n*-dimensional raster data^28^. In the 3D data, the foraminifera are separated from the filling medium based on a voxel intensity threshold. This threshold was found empirically and tuned to strike a balance between preserving foraminiferan material whilst completely removing the filling medium. After checking the resulting disconnected components for segmentation faults, the individual specimens are exported as .nrrd files. This segmentation procedure is implemented in another Python script (segment.ipynb, see Code availability).

### Further expansion

ForametCeTera is a diverse 3D dataset yet further expansion would enhance the robustness and generalisability of (machine learning-based) classifiers. Diversity can be expanded by scanning specimens from different species, regions and depths. Additionally, scans of *unprocessed* samples would enable the testing of trained classifiers on real-world data that was minimally pre-processed as envisioned in the proposed procedure (see Fig. 2).

## Data Records

**Fig. 5:**
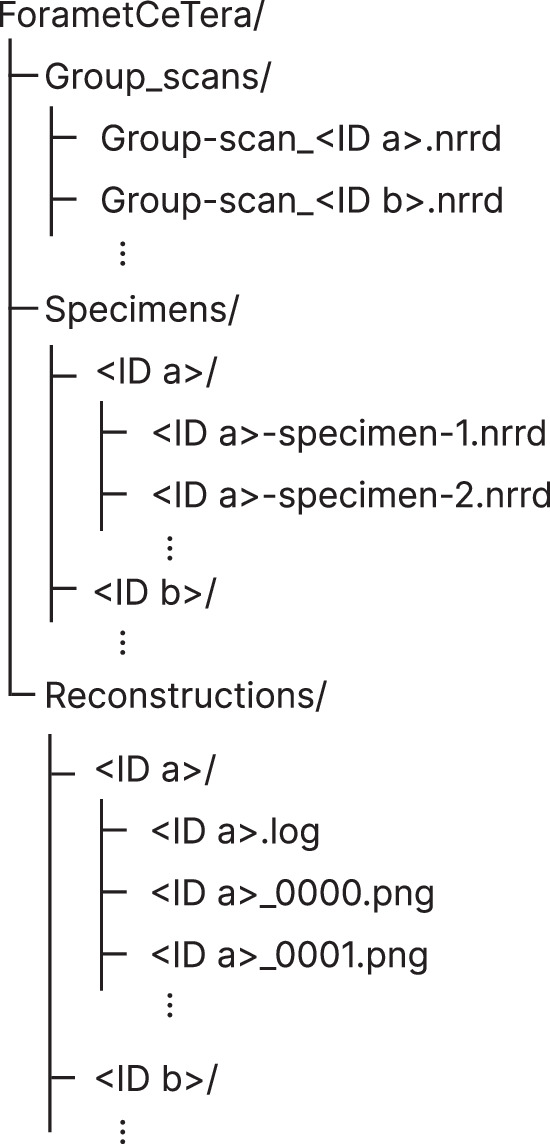
The dataset structure of ForametCeTera.

ForametCeTera is publicly available as a .zip file at Zenodo^29^ of about 4.4 GB. This file contains the 2D reconstructed group-scan cross-sections, the 3D group-scan data and the individual, segmented 3D specimens. The raw projection images of the group-scans are available upon request as the data is relatively large (332 GB uncompressed). An overview of the data and its metadata is given in Tables 1 and 2. The way the dataset is structured is shown in Fig. 5. The Group_scans folder contains the 3D reconstructions of the group-scans, the Specimens folder contains the segmented 3D specimens i.e. the segmented group-scans, the Reconstructions folder contains the reconstructed 2D cross-sectional data and a file output by Neoscan80 on the scanning parameters per scan. Specimen indices in the Specimens folder may jump due to touching specimens that were erroneously segmented as one and subsequently not exported. For the sake of reproducibility, the indices were not corrected for this. <ID a> placeholders refer to the IDs as shown in the Dataset ID column in Table 2.

## Technical Validation

The Neoscan CT scanner undergoes regular maintenance and calibration. Prior to each scan, the flat-field reference is updated by the Neoscan software. The reconstructed 3D data has been examined to identify any oddities in the captured intensity values. The segmented specimens have been checked on intensity oddities and on segmentation faults.

## Usage Notes

The 3D data can be viewed using, for example, the open-source program 3D slicer (https://www.slicer.org/). To perform analysis, Python was used in conjunction with several packages like pynrrd to load the data and numpy, scipy.ndimage and scikit-image to perform further analysis. For machine learning endeavours, the TorchIO or rising (exclusive to PyTorch) libraries may be used.

## Data Availability

All code used in this paper is available at https://github.com/JLuij/ForametCeTera_scripts. This includes the script to convert a stack of .png cross-section images to an .nrrd file (stack.py), the script for segmenting the group-scan volumes (segment.ipynb) and the script for performing the technical validation (technical_validation.ipynb). As these scripts make use of several packages, an environment.yml file is included to reproduce the conda environment, together with more instructions in the repository’s Readme.md file.
